# Hard talk, costly walk: The evolution of a soft budget constraint syndrome in a football club at the periphery of Europe

**DOI:** 10.3389/fspor.2023.1107988

**Published:** 2023-03-07

**Authors:** Bernt Arne Bertheussen

**Affiliations:** School of Business and Economics, UiT The Arctic University of Norway, Tromsø, Norway

**Keywords:** hard budget constraint, soft budget constraint, budget rhetoric, benefactor, financial fair play regulation

## Abstract

A football club is exposed to a soft budget constraint (SBC) syndrome if it still survives after finding it impossible to break even financially in the long run. This in-depth case study presents a historical analysis of the evolution of an SBC syndrome in a top-tier Arctic football club over 30 years using public archive data. In oral speeches, strategy documents, and other official situations, the hierarchy at the club emphasized the importance of operating in a financially sustainable manner, that is, complying with a hard budget constraint approach (HBC). The club went along this path during the first years after entering the top tier in the mid-1980s. This was made possible by a team of low-cost local players. However, in line with increased media and sponsorship revenues in the 1990s, the economic threshold for competing at the top-level rose. Thus, during the last two decades of the club's financial history, the budget constraint (BC) approach softened. Primarily, a distant benefactor and capital injections from a joint-stock company owned mainly by the local bank and local energy company funded the overspending. Furthermore, the study uncovers how a soft financial control and monitoring system introduced by the national football association failed to emancipate the club from the SBC syndrome. The study finally suggests some measures that can eventually harden the BC at this as well as many other financially struggling European football clubs.

## Introduction

Financial problems in European club football have been long-lasting (1–5). However, the implementation of European and national regulations has curbed some of the spending and helped clubs compete under more balanced financial conditions (6). For the top clubs in the big five leagues (England, France, German, Italy, and Spain), increased media revenues have also contributed to fewer deficits in the accounts. In the non-big-five leagues, the clubs are still struggling with major financial problems. The same is true for the clubs below the top tier of the big five, especially in the English Championship (7). The average revenue per club in English Premier League was in 2018 almost 300 million euros. The corresponding figure for a small league such as the Norwegian one was 10 million euros (6). The financial gap between powerful and poor clubs is, therefore, substantial.

For a football club, the primary strategic choice is to be made between profit and win maximation (8). There is a consensus in the sports economics literature that win maximization seems to be the objective of European clubs (9–13). Haugen and Solberg (14) illustrate that European clubs can end up in a dilemma because there is substantial pressure on them to overspend to pass needle eyes, that is, to win the league, avoid relegation, acquire promotion, or qualify for lucrative European tourna­ments.

However, in recent years state-owned football clubs and sports washing has become a more prominent strategy in European football (15). We use the term sport washing to describe the practice of individuals, corporations, or governments to improve dubious reputations. Accordingly, sport washing is a form of propaganda. Late in 2021, a Saudi Arabia-led consortium headed by the Saudi sovereign wealth fund purchased Newcastle United. Newcastle United is not the first Arab takeover of a European football club. Wealthy Arab owners now own top clubs like Manchester City (United Arab Emirates), Paris-Saint Germain (Qatar), Aston Villa (Egypt), and Sheffield United (Saudi Arabia). Arabian aviation companies like Emirates, Etihad Airways, and Qatar Airways all have signed deals with top European football clubs.

Storm and Nielsen (16, 17) argue that European clubs operate in a regime with flexible or soft budgets. In the local community, the club has characteristics of a social institution through its relationship with key stakeholders, primarily the supporters. For them, the club is a vital identity marker (18) and considered too important to fail (19). Consequently, one or more stakeholders will bail it out once the financial problems seem insurmountable (20). The club is, therefore, likely to receive free cash from an owner, creditor, or benefactor to stay liquid and in business. The subsidies can, for example, come in the form of equity without normally required return, loans with abnormally favorable interest and installment terms, donations, overpriced sponsorship contracts, and underpriced stadium rent to make the budgeting constraint (BC) of the club softer (21). However, a soft BC (SBC) undermines one of the most essential financial coercive drivers for a firm in a market economy, that is the threat of bankruptcy (22). The syndrome, therefore, encourages clubs to take excess risks, downplay the significance of good governance, and allow the emergence of bad management practices (21).

This archive-based in-depth case study mapped and analyzed the financial intentions (talk) and behavior (walk) of the case club, Tromsø IL (shortly named TIL), and its close allies, that is, its environment. The objective was to gain deeper insight into the antecedents and consequences of an SBC syndrome. During the period studied, the club and its allies made several momentous interventions that shaped the transformation of its BC syndrome. The club gained promotion to the top-tier in the national football league in 1986 with local players on a minimum budget. During the approximately first ten years that followed, the club experienced sportive success with small funds under an HBC syndrome. The league's cash flow then gradually increased in the form of more significant media, sponsorship, and gate receipts revenues. As the financial threshold for competing at the top level was raised, the club's BC syndrome softened. The softness was further reinforced by a relegation and subsequent promotion in the 2001/02 seasons.

In the years that followed, the club again experienced great sportive success, now supported by financial injections from a benefactor. However, in 2008, the finances collapsed again. To solve the problem, the commercial part of the club was reorganized into a hybrid sport/business enterprise—the so-called Dual Model (23, 24). The intention was to eliminate the financial problems once and for all through increased professionalization and commercialization of the top football operation. However, the added cash flow that followed did not make the BC harder but even softer as it enabled the overspending to continue. In 2011, the national football association (NFA) realized that Norwegian clubs could not manage their finances by themselves and introduced a variant of a financial fair play (FFP) control and monitoring system. Despite good intentions, this system had inherent weaknesses that allowed the club to continue overspending, though only to a certain extent.

In particular economics, the influence of the SBC approach has been significant (25). This will also become evident in the literature review of this paper. This article aims to expand on existing literature as the SBC field of research is still in its initial stages in the area of sports economics and management (26).

Moreover, in the SBC literature, there have been, to this point, few in-depth case studies that explore the evolution from an HBC to an SBC syndrome over a long period of time. This paper argues that to dampen the financial problems of European football, a better understanding of why an SBC syndrome occurs, how it occurs, and what characterizes its development is crucial. Accordingly, the following research questions are raised in the present study:

*RQ1: Which budget constraint syndrome was advocated by the hierarchy of Tromsø IL?*

*RQ2: What characterized the financial and administrative budget interventions made by the club and its allies, and how did the interventions impact the budget constraint syndrome of the club?*

This case study thus explores the evolution of an SBC syndrome at a specific football club, Tromsø IL (TIL). The case club is exceptional in its unique geographical context with a cold and harsh arctic climate. Nevertheless, TIL is the northernmost professional football club worldwide to have won national trophies (27). It may be of particular interest to study the financial management of a football club that has to operate in such an extreme environment as it may require special measures to succeed in sport and at the same time survive financially. SBC theory has not previously been applied to this specific geographical, climatic, or population-wise context. Storm et al. (26) argue that more case study evidence of SBC is needed. They further state that, in particular, more case studies should be applied to new empirical context such as countries.

The paper proceeds as follows. A literature review is provided first. Second, the historical context of the club is described. Third, a chapter on methods and data follows. Fourth, findings are presented and discussed consecutively, and finally, a discussion of the contribution of the results ends the paper.

## Literature review

A professional football club that aims to be financially sustainable must break even over the course of a few years (28). To do so, the club must limit its spending on player wages to finance these and other operational costs through current revenues (29). If this is the case, the club controls its finances through an HBC syndrome (30). A club that does not break even over time will experience recurring deficits. Consequentially, its finances are regulated through an SBC (16). The club can finance deficits through equity issues or donations from benefactors (31). The remaining option is loans if these capital sources are unavailable or insufficient. Net debt will occur in the club's balance sheet if its liabilities exceed its assets. However, after some time, the net debt accumulation may not be economically sustainable (32). The club will then be at risk of becoming insolvent, illiquid, and eventually bankrupt.

A European football club rarely goes bankrupt despite ongoing financial problems (20). When it is on the brink of bankruptcy, an insolvency process is avoided through a bailout. The club is regarded as too important a social institution in its local community to be allowed to fail (19). Institutionalized bailout practices have added fuel to the never-ending spending race. Overspending gives a club a financial competitive advantage over rivals that do not adopt a similar strategy (6).

Storm and Nielsen (16, 17) use the SBC theory to explain why most European football clubs survive despite persistent deficits and growing debts. They argue that many clubs are similar to banks and financial intermediaries as they may be regarded too *big* to fail by the authorities. In a football context, though, it may be that other stakeholders believe that the club is too *important* to fail. Among these are fans, TV viewers, sponsors, benefactor, investors, local government, tax authorities, local banks, or any other benefactor (21). Individually or together, these stakeholders can be willing to inject the cash flow required to rescue a club in financial trouble or help a club buy an attractive player that it could not afford otherwise. According to Andreff (21), this is precisely what the SBC syndrome is about. From a club's point of view, the management can feel reasonably assured that one or more external stakeholders will step in and bail the club out when needed.

The presence of the SBC syndrome in an industry can be measured by the frequency of deficits, bailouts, and bankruptcies (30). Thus, Franck (32) claims that the SBC syndrome results in detrimental managerial incentives and moral hazard in that overspending and recurring deficits have no consequences for the decision-makers. He thus argues that the SBC approach encourages the “financial doping” of football clubs; that is performance-enhancing social practices (33, p. 73). Storm and Nielsen (17) underline that clubs that do not focus on balancing the books will pay less attention to efficiency and waste scarce financial resources. Terrien and Andreff (34) support this argument.

Kornaï (35) lists soft ex-post bargained subsidies, soft ex-post bargained taxation, soft ex-post bargained credit and soft administrative pricing with authorities as different ways to soften a club or firm's BCs. In some cases, SBC support may be considered an effective political strategy to get more voters (36). When politicians support their local football club through some form of subsidy, they can strengthen their reputation among supporters and fans who can form a large constituency at the next election.

If many clubs mimic each other's soft financial behavior, this will create an excess demand for critical input, such as player talent (6). A subsequent pay pressure in the industry will then occur as clubs overbid for talent and engage in an arms race for input that is in short supply. This is especially true for superstars (37). A club's ability to buy players financed by a benefactor or other supporting stakeholders can dramatically expand its demand for talent. This can, in turn, lead to shortages and increased transfer prices. Accordingly, the SBC syndrome encourages the propensity to overinvest in players.

Kornaï et al. (38) claim that a true SBC syndrome is at work only if organizations can *expect* to be rescued from financial problems. Such expectations will create an SBC mentality in the club's management team. The SBC syndrome occurs if the club's management knows, guesses, or is beforehand aware that one or more supporting stakeholders will repeatedly and sometimes unwillingly cover all or part of the deficits or past due debt (21).

Kornaï (39) claims that an HBC is characterized by economic coercion where “proceeds from sales and costs of input are a question of life and death for the firm” (p. 303). BCs are also hard if the firm's growth depends on its profits or what it can receive from conservative owners and lenders only for profitable investment purposes (17).

SBC syndromes, manifested through ongoing overspending and subsequent bailouts, have been ongoing for decades in European football (16, 21). The two interrelated and self-destructive economic practices constitute a vicious financial circle (40). Bertheussen and Solberg (6) argue that a dominant sportive and a subordinate financial institutional logic underpin the practices. These conflicting logics form a Gordian knot, which the clubs themselves cannot cut off, as they strive to gain a competitive advantage over their rivals by paying more in transfer fees and player salaries. The relative competition is, however, a zero-sum game. If one club improves its table position, this will always be at the expense of another (41). Rivals responding to this pay game through mimetic behavior will make the wage level throughout the league increase without anyone having achieved a sportive advantage. Thus, a strategy that initially seems rational for the individual club can later turn out to be irrational overspending for the industry (42). Industry regulations are required to cut this Gordian knot (6).

**Figure 1:**
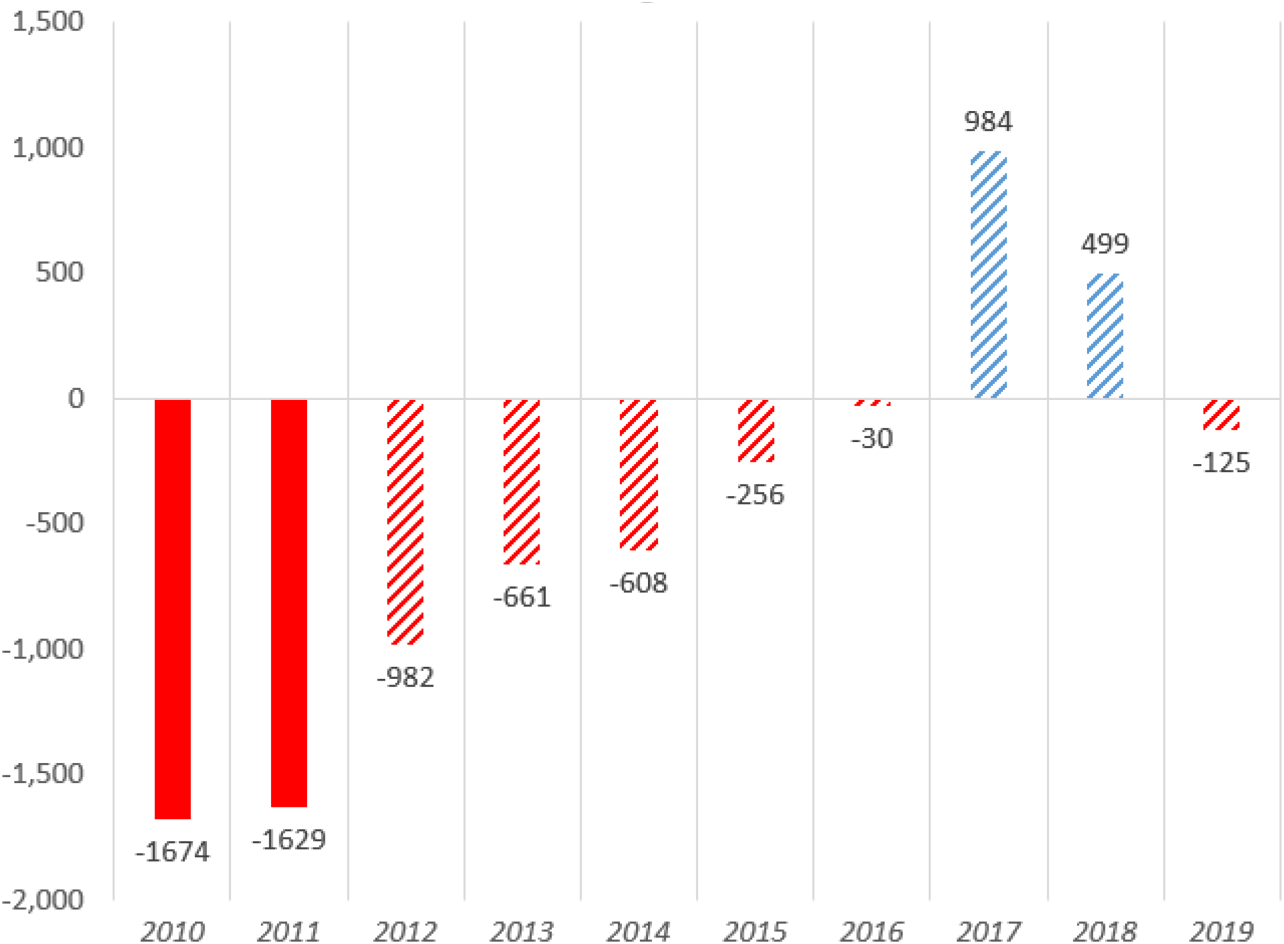
European club’s bottom line losses and profits (million euros) before and after the gradual introduction of FFP in 2012. Source: Deloitte (44).

UEFA's Financial Fair Play (UEFA FFP) regulations introduced in 2011 aim to tackle the problem of clubs that do not themselves manage to break even. This is thus an attempt at hardening the BC (29). UEFA FFP was intended to curb clubs’ spending and help them compete under the most comparable financial conditions. Its purpose is to reduce overspending and make the clubs’ finances more sustainable (28). Furthermore, several domestic leagues have introduced club licensing schemes based on criteria similar to the UEFA FFP regulations (43). After implementing financial regulations at both national and European levels, a significant improvement has been observed in club finances across Europe (see Figure 1) (44).

However, many small and medium-sized clubs are still struggling with their finances. The regulations introduced are therefore criticized for being too soft (45).

Andreff (21) argues that what is wrong with UEFA FFP is its tolerance of an annual €5M deficit, as wealthy clubs can take advantage of this threshold and distort sportive and economic competition to the detriment of clubs that do break even. Andreff further asserts that UEFA FFP has not gone far enough in fulfilling HBC that is not distortive. This will require that the break-even rule is strictly adhered to as for businesses in general in market-based economies.

Other aspects of FFP have also been criticized in the literature. Drut and Raballand (46) discuss the impact of FFP on the quality of all teams and argue that financial regulation is critical to explain performance differences in football competitions. Their empirical findings from the big five leagues show that clubs allowed to operate with red numbers hired better playing talents and obtained better sportive results. Franck (29) argues that FFP prevents substantial injections of external financing and thus facilitates a fairer competition arena. Taking a game theoretical approach, Preuss et al. (45) examine the impact FFP could have on player wages. They found that the clubs had strong incentives to bypass the new regulations. Sidestepping would, however, cause extra costs for clubs to hide a violation of rules and for UEFA to expose dishonest behavior. These added costs might deter smaller clubs from cheating and thus provide a competitive advantage for larger clubs. The impact of FFP on the competitive balance of domestic leagues is discussed by Plumley et al. (47). They found a significant decline in the competitive balance after FFP was introduced in France, Germany, and Spain but not in England and Italy. Syzmanski (48) questions the legality of the regulations. He argues that the objectives of FFP are not fairness but financial efficiency. He further claims the rules introduced are unable to improve efficiency.

In 2022, UEFA's FFP was replaced by new Financial Sustainability Regulations (49). UEFA justifies the new regime by stating that sustainable financial management has become even more important for the clubs in the wake of the COVID-19 pandemic, increased globalization, and technological innovations. According to UEFA, the new regulations will ensure that the clubs must be stable, solvent, and keep their costs under control. The new regulations consist of three distinct pillars. The no overdue payables rule tightens the clubs’ requirements to pay outstanding debts. The football earnings rule is a modified version of the Break-even rule. The squad cost rule, which is new, restricts spending on player and coach wages, transfers, and agent fees to 70% of club revenues.

## The club's environment and identity

The Arctic climate is characterized by long, cold, dark, and snowy winters. Thus, TIL accrues high costs in the winter for electricity and to clear the pitch from snow. In the summer, however, the sun shines 24 h a day, and life is easier. The gentle Gulf Stream flows along the coast and keeps it ice-free year-round. The long coastline is rich in fish, and the spawning migration of cod from the Barents Sea to the coast from January to April has historically formed the basis for the coastal settlement.

Consequently, there is a saying up north “In cod, we trust.” The club's strategic plan document (2017–2020) explicitly states that player recruitment shall reflect the harsh Arctic climate, and players raised locally should therefore be prioritized. The document also states that the club's playing style shall be offensive in line with the optimistic temperament of people living in the Arctic. The formally expressed strategic vision of the club is, in this way, closely linked to the identity of the locals.

When the club plays an away derby match, the supporters must travel about 50 min by plane, 530 kilometers by car, or 27 h on the coastal line boat. TIL was founded in 1920, and its stadium Alfheim is named after the residence of the Norse god Freyja. It has a capacity of some 7,000 spectators. Recently, on match days, it has hardly been half full. The club's best historical table positions are two second places in the top national league in 1990 and 2011.

Furthermore, it has twice been the national cup champion (1986 and 1996). The national cup trophies make TIL the northernmost club in the world to have won a national title (27). Additionally, TIL participated in the group play of the Europe League in 2009, when Atletico Bilbao narrowly beat it, and in 2013 the club participated unsuccessfully in the same competition. In the 2019 season, the club was relegated to the second level; however, the following season, it was again promoted to the top tier.

When this case study started about 1985, TIL was a pure membership club. In 2009, the club structure expanded and became part of a group of cooperating joint-stock companies. The companies were TIL Fotball AS, TIL Spiller AS (“Spiller” translates to “Player” in English), and TIL Holding AS (“Holding” translates to “Group” in English). TIL Spiller AS was eventually terminated during this long-term case study, and so was TIL Holding AS.

## Methods and data

The present case study explores the economic history of the Norwegian football club TIL. More precisely, the study delves into the how's, why's, and what's of the evolution of an SBC syndrome at this specific club. Thus long-term data was needed, and a historical in-depth case study research design was selected.

Case studies are rich empirical descriptions of a phenomenon based on various data sources (50). The basic idea is to use one or more cases to develop theory inductively (51). In case studies, the theory is emergent because it is situated and developed by identifying patterns or relationships among constructs and their underlying logical arguments.

A strength of case study research is its ability to integrate objective and perceptual data (52). This study used document analysis (53) to provide relevant data on the relationships between the intended vs. realized BC syndrome at a professional football club. The study integrates and triangulates several sources of secondary data for the period between 1986 and 2019. This involves the club's strategic business plan documents, annual reports such as the board's report, financial statements, reports from the Control Committee, and reports from the NFA about the club's financial status and development (the national FFP report). Moreover, an investor prospectus was presented in 2009 when the top football part of the club was converted from a membership organization to a joint-stock company. Finally, the study has followed industry media such as newspapers and web pages to obtain additional relevant information.

Data were gathered and analyzed concurrently by data reduction, collecting key information in sections based on topics of interest. The data amount was voluminous, as often is the case of qualitative data collection. It was, however, necessary to go in-depth as this study's theoretical contributions are being nourished through thick descriptions (54) of the club's SBC interventions (see next chapter).

## Findings and discussion

The findings of the study are presented and discussed consecutively and separately. The interplay between the results will finally be addressed in the Overall discussion chapter.

### Budget constraint rhetoric

The first research question (RQ1) in this study was, *Which budget constraint syndrome was advocated by the hierarchy of TIL?* In response to RQ1, the extracts from the Investor prospectus were reproduced and compared with the information presented in the club's Internal Control Committee's (ICC) report the same year (2009).

#### Promising a bright HBC future: the investor prospectus

In 2009/10, the most comprehensive strategic intervention in the club's history took place as the commercial and professional part of the club was transformed into a limited company structure (TIL Fotball AS and TIL Holding AS; also see Intervention 2: The hybrid organization model). The new Model worked alongside the original club membership organization. Table 1 presents excerpts from the Investor prospectus.

**Table 1:** Excerpts from the investor prospectus of 2009/10*.

The company's purpose is to promote the sportive development of the club and secure profitable operations. The club aims to steadily position itself among the top-six teams in the top-division table. The club considers the whole of northern Norway as its local market and recruiting region. Emphasis will be placed on recruiting regional players, and the club should be the natural first choice for the region's most promising football talents. The talents will be well taken care of and refined, and the goal is to have an as high proportion of players from northern Norway as possible. The local anchorage will contribute to pride, better audience support, and a more extensive and better market for commercial exploitation of the brand represented by the club. Greater emphasis on local recruitment from the region and further enhancement of the talents will contribute to more significant revenue from the turnover of player sales. The board of the club's commercial holding company considers the region of the city, northern Norway, and the entire north area (including Norway, Sweden, Finland, and Russia) as a potential future commercial market. During 2010/11, the board will develop commercial strategies that cover the entire northern region.
Achieving the sportive goals will help TIL Holding AS through its commercial activities based on the subsidiaries TIL Fotball AS and TIL Spiller AS to be able to achieve, in relation to risk, a reasonable return on invested capital through an increase in the value of the shares and eventually through dividends to the shareholders. Other financial goals are to reach positive operating results for the top football business and gains from player sales.

* Author's translation.

#### The club's control committee raises the SBC red flags

The ICC is part of the club's governance structure, tasked with overseeing the efficiency of the club's operations, the reliability of financial information, compliance with laws and regulations, and the protection of the club's assets. Table 2 thus presents excerpts from the ICC report of 2009, the same year TIL Fotball AS and TIL Holding AS was founded.

**Table 2:** Excerpts from the report of the control committee in 2009*.

‘The club presents a financial statement for the 2009 financial year with a profit of Norwegian kroner (NOK) 16.4M from a turnover of NOK 108.3M. However, if donations and capital inflow from the sale of rights are excluded, the club would have lost NOK 27M from a turnover of NOK 63M. The club's operation is thus very poor because it has spent significantly more money than it earned. The value of the club's assets during the year was lower than the debt, and the club has not been able to fulfill its obligations when due. Technically, the club is insolvent, and it would have gone bankrupt had it not been for donations and capital injections. Although the club's financial position seems to have been saved through adding capital toward the end of 2009, the Control Committee notes that the amount collected has to its full extent, only covered accumulated deficits. In other words, funds are not available if operations were not balanced in 2010.
The very significant operating deficit is, to a large extent, related to the lack of player sales. The 2009 budget included player sales of NOK 15M. Even though during 2009, bids were received for players who more than fulfilled this budget item, the board has deliberately chosen not to sell players. The committee assumes that the board was familiar with the club's financial situation at the time when it was possible to sell players. In this regard, the board has not followed the mandate given to them by the annual meeting. The Control Committee finds this criticism noteworthy, especially because similar circumstances were pointed out in the committee's report for 2008.
… the committee will also underline that the club's general salary level is too high, even though significant work has been done during the year to adjust it. There are a few employees in the sports department who burden the club's salary budget with a disproportionate share. … The club's management could and should have taken more steps than it has to reduce these expenses.
… through auditing reports, it has been revealed that the club's accounting department is understaffed. Thus, the Control Committee recommends strengthening this function significantly. Not only does lousy accounting entail significant additional expenses related to auditing, but it also deprives the club's management of its most important management tool in the financial operation of the club.
… control reports have also revealed a worrying lack of professionalism related to the signing of financially significant agreements. On the one hand, agreements exist only orally as so-called gentleman's agreements; on the other hand, they consist of handwritten notes. This has caused the club to lose significant financial rights.

* Author's translation.

#### Discussion of the budget constraint rhetoric

Several statements in the prospectus presented in 2009/10 (see Table 1) indicate that the club intended to operate within HBC principles. Targeting of profitable operations is made explicit in the very first sentence of the prospectus, and potential investors are informed that they can expect a reasonable return on invested capital relative to risk. This is another strong HBC statement followed by more detailed financial objectives of obtaining positive operating results and gains from player sales. Based on the well-defined financial goals in the prospectus, it seems reasonable to conclude that the intention of the club (the talk) was to operate in line with HBC principles when it was transformed into a hybrid organization or Dual Model in 2010 (for more on this Model, see Intervention 2: The hybrid organization model). Thus, the statements in the prospectus suggest that the SBC approach hereafter should be considered a sin of the past. Unfortunately, the club's walk, which the study will focus on next as a response to RQ2, tells a different story.

The Control Committee underlined in 2009 the soft reporting of the club's financial results (see Table 2). The committee pointed out that the reporting does not reflect the outcome of normal operations, as both turnover and profit are strongly positively influenced by extraordinary events. A profit of 16.4M NOK was reported, whereas the result of normal operations showed a deficit of 27M. The board was also criticized for not selling players according to the annual meeting mandate, representing the club's highest governing body. The committee further underlined that there were a few players with a disproportionate share of the club's salary budget, that the accounting department was understaffed, and that there was a worrying lack of professionalism when entering into significant financial agreements.

To sum up, this study finds it reasonable to conclude that the Control Committee strongly criticized the board for following an SBC approach in the club's operation. The committee urged the board to harden the BC approach at the club. However, the Control Committee has no decision-making power in the club's governance. This is merely an advisory body that gives a written statement of the club's overall operations to the members at the annual general meeting.

### Soft budget constraint interventions

This chapter is a response to RQ2 of the study: *What characterized the financial and administrative budget interventions made by the club and its allies, and how did the interventions impact the budget constraint syndrome of the club?* Characteristics of the implemented financial interventions and their impact on the BC syndrome of the club will be discussed one by one in this chapter.

The first soft intervention was introduced in 2006 and represented a generous distant benefactor who has provided the club with more than 120M NOKs in donations since then (27). The second soft intervention discussed integrated the membership club's operations with a new joint-stock company structure in 2009. The membership organization and the limited company jointly constituted a hybrid or so-called dual organization model (23). This Model is the most common form of organization among the top teams in the nation involved (24). The third and final soft intervention addressed a national FFP system (hereafter national FFP in contrast to UEFA's FFP) aimed at curbing club overspending. This system was imposed on all professional clubs of the top-two tiers in 2009. This external control and monitoring intervention can be interpreted as an NFA attempt to create a counterweight to the drift toward developing SBC syndromes at most professional football clubs in the nation.

#### Intervention 1: benefactor support

##### Characteristics of the benefactor intervention

A national finance magazine's overview of the wealthiest persons in the nation showed that the benefactor was in top 20 on the list with more than 10 billion NOKs (10 NOKs equal about 1 EUR) in wealth in 2018 (55). Within football, a number of clubs have been receiving funding to a greater or lesser extent from the same benefactor, including his local club.

##### Impact of the sugar daddy intervention on the budget constraint syndrome

In the local media interviews, the benefactor underlined that he regarded himself as a financial security for those who manage the club (Table 3, Subpoint 1). He further thought the money was well spent (Table 3, Subpoint 2). According to Table 4, the distant benefactor has provided the club with close to NOK 140M since 2006. A number of projects have been catered for, including bailouts (101M), talent development (18M), and infrastructure investments (17M).

**Table 3:** Benefactor motivation and expectations (excerpts from local media interviews)*.

	Theme	Excerpt and Comments
1	On providing financial security for the management of the club	When the journalist asked what he thought about how important he was to the club over the years, he was a little uneasy. “I think my role has been exaggerated, but I have probably been financial security for those who manage the club. It's good to know you have someone to rely on,” he answered.
2	The benefactor's assessment of how well the club has spent his money	“Running a football club is not an easy venture. All the time, you have the dilemma between short-term and long-term investments. But I think the academy at the club has done and still does a respectable job in developing its players. Getting good internal recruitment is an excellent model. It's money well spent,” the benefactor said.
3	Relation to the club and the fans	The benefactor was born and lived on the west coast in the next largest city of Norway. According to Google Maps, the distance between the two cities is 1,824 km by car, with an estimated driving time of 28 h. “I’m supposedly the only westerner to be welcomed at TIL's stadium, at least according to the ‘Iceberg’ (nickname of a hardcore fan group),” the benefactor said to the newspaper while sitting in one of the lodges in the stadium building, he owns and rents cheaply to the club.
4	On the governance of the club	The benefactor applauded the club's plan to have one joint board for the club and the corporation instead of two, which has been the case for the past ten years. “I think it makes sense, and it will be easier for the managing director to serve one boss instead of two.”
5	On player recruitment as of March 2019	As of today, there are no players on the way using funds from the benefactor. “I haven’t heard of anything, and I don’t think the club has either. They want to work with the players they have,” the benefactor said.
6	On recruitment of local academy players	The benefactor is more concerned that the club is giving homemade and local players a chance. “It is important that players from the academy get a chance, and maybe not the whole season because, as the coach told me, the young players will vary in performance. It's enjoyable when local, young players succeed,” he added.
7	On following the club in everyday life	”I think about them all the time and watch some TV matches. In addition, I talk to my local adviser up north three times a week.”
8	On the loyalty of his hometown club and TIL	“It gets ‘complicated’ when my two teams meet, and my only comfort is that one of them will get the points. If it becomes a win and a loss for each club, as it was last year, then that's fine for me. The points are then evenly distributed,” the benefactor said.
9	On loyalty to TIL	The benefactor has been involved in the club both when the team has been near the top of the table and when it has been relegated. “We who are the loyal type naturally follow along,” he said.
10	On establishing a benefactor community	The benefactor's advisor up north promises that money will be made available to new players in the coming months if others are willing to share the bill. “We are appealing,” the advisor adds, “but it is important that there is a collaboration of three equal parties, I think.”
11	His engage­ment in processes lead­ing to player investments	“ I have contributed to some player purchases and just said yes. After all, I know nothing about which player types to buy. It must be up to the coach,” he said. He further pointed out that everything “doesn't have to take that long.” He added, “When my advisor and I decide something, it often takes about 1 min.”
12	The motives behind the donations	Where does this desire to give come from? “I think it's something you must have; either you have it or don’t have it. For my part, I think it comes from when I was a small boy. Whether one is willing to share soft drinks or sweets with your siblings, for example.”
13	On the duration of the engagement	The answer was a resounding no when asked if he should step down from his commitment to the club.

* Author's translation. Sources: iTromsø, Rune Robertsen, June 7, 2018, and March 23, 2019 (56).

**Table 4:** Intervention impact: examples of donations given (all values are in MNOKs)*.

Year	Purpose	Category	Value
2006	New artificial turf on home ground	Infrastructure	7
2009	Donation to the club	Bailout	10
2009	Contributes to establishing a joint-stock company structure	Bailout	15
2010	New artificial turf on home ground	Infrastructure	5
2011	Donation to the club	Bailout	10
2012	Donation to the club	Bailout	10
2013	TIL is at risk to be put under administration by NFA	Bailout	5
2014	Money earmarked player development	Talent development	5
2015	TIL receives 10 million over 3 years for talent work	Talent development	10
2015	Buys premises in the stadium and rents these back to the club cheaply	Bailout	38
2016	Pays 2 million for renovation of stadium	Infrastructure	2
2016	Gives TIL's A-team a commitment of 5 million	Bailout	5
2017	Donation of 3 million for development work	Talent development	3
2018	Contributes with salary to two players	Bailout	1
2018	New artificial turf on home ground	Infrastructure	3
2019	Cost-sharing (about 50%) with other benefactors	Bailout	7
	Bailouts		101
	Talent development		18
	Infrastructure		17
	Grand total		136

* Authors translation. Source: https://www.aftenposten.no/sport/fotball/i/AdR17M/har-gitt-120-millioner-paa-12-aar-aapner-for-aa-bruke-nye-mohn-penger-paa.

##### Discussion of the sugar daddy intervention

To compensate for overspending is an archetypical feature of an SBC syndrome (30). The primary sources of soft funding in this specific case study were a distant benefactor. This study has no reason to believe that the benefactor did not have all the best intentions with his contributions, as he wanted the club to be a proud representative of its region. Nevertheless, from an SBC perspective, the most critical mean of getting rid of overspending is to stop financing it (6).

#### Intervention 2: the hybrid organization model

##### Characteristics of the hybrid organization model intervention

A football club needs to gain a license from the football association to participate in league and cup competitions. However, the NFA only issues licenses to member clubs, not to joint-stock companies. This arrangement contrasts with most other European countries (57). However, a club can have a formal partnership agreement with a joint-stock company (23). Together, they constitute a hybrid organization or a Dual Model. Still, only a club can be awarded a license. In addition, all players must be employed formally by the club. Thus, the club must pay salaries to the players. Therefore, the consolidated accounts of the club and the joint-stock company form the basis for the club's license. These consolidated accounts also form the basis for the club's score in the FFP control and monitoring system, shortened FOS. The purpose of the Norwegian hybrid organi­zation model is that the club should retain control of the sportive activities even after it has entered into a formal agreement with a limited company structure that controls the cash flow of the football business activities (23).

TIL established a complicated group structure in 2009, which consisted of three limited companies. The primary responsibility of TIL Fotball AS was to exploit the market potential of the club and take care of the daily business activities. TIL Fotball AS should, in turn, annually provide the club with an amount corresponding to the salary costs of the players. TIL also established a third-party player company TIL Spiller AS. Player rights, including player valuations, were transferred to this company. Finally, TIL established a third joint-stock holding company: TIL Holding AS. This was the parent company of the two joint-stock subsidiaries of TIL Fotball AS and TIL Spiller AS. The local savings bank, the local energy company, and the club owned about 23% of the shares each in TIL Holding AS (27). Finally, the club's benefactor gifted NOK 15M to establish the company structure. The Dual Model or the hybrid organization structure is now established at the world's northernmost top-level football club (58). Table 5 provides an overview of the economic development of the top football activities of the enterprise before and after the introduction of the Dual Model.

**Table 5:** Economic development before and after the hybrid organization model*.

TIL, a Membership Organization					
1986-1995: The club mostly broke even except in 1992, when the deficit was 2.3M NOK.					
1996-2000: The club managed to finance large operating deficits with player sales, and players were sold for over 90M during this period. In the year 2000, for example, the turnover was NOK 19M, but with player sales of 28M, it ended at 47M. This year's profit of 16M was the largest in the club's economic history.					
2001-2005: In the relegation year 2001, the deficit was NOK 8M. In the following promotion year, the deficit increased to 11M before falling to 5, in 2003. The club made a profit in 2004 and broke even in 2005.					
	Revenues	Operating Profit	Donations^1^	Normalized Operating Profit	Normalized Operating Margin
2006	48	−10		−10	−21%
2007	65	0	7	−7	−10%
2008	68	−14		−14	−21%
2009	64	−12	15	−27	−42%
Average	61	−9	6	−15	−24%
TIL Fotball AS, a Joint-stock Company					
2010	57	−20	5	−25	−43%
2011	74	1	10	−9	−12%
2012	80	−5	10	−15	−18%
2013	91	1	5	−4	−5%
2014	53	−4	5	−9	−17%
2015	48	−6	10	−16	−34%
2016	49	0	7	−7	−15%
2017	41	−8	5	−13	−33%
2018	43	10	11	−1	−3%
2019	44	2	15	−12	−28%
Average	58	−3	8	−11^3^	−22%

* Sources: 1986–2005; media coverage in local newspapers. 2006–2009: Investor prospectus of TIL. 2010–2019: yearly financial statements of TIL Fotball AS.

^1^ Only cash donations are included in this column. The donations the club has received in saved annual rents since 2015 have not been included, even though the benefactor bought parts of the stadium for NOK 38M and since then has rented the same premises cheaply to the club.

^2^ The shares from the local savings bank and the local energy company, estimated to be 22M NOK, that the club received free of charge in 2018 had no cash effect and are thus not included in the Donations column.

^3^ Aggregate normalized operating profit adds up to 111M for 2010–19. This is a slightly higher figure than what the club itself states in the financial statement for 2019 as the accumulated carry-forward deficit of 104M accrued in 2010–19.

##### Impact of the hybrid organization model intervention on the budget constraint syndrome

When the club was first engaged in top-tier football in 1986, the side was dominated by low-cost local players (27). Table 5 shows that during the first decade at level one, the club essentially broke even and thus followed an approximate HBC approach. In 1989, the club finished second and then third in the national league competition. From the mid-1990s, the cash flow to the Norwegian football industry has increased due to more audience, media, advertising, and sponsorship revenues. Higher revenues led to more competition for the best players and increased salaries (59). Thus, the club became dependent on selling local talents to make ends meet. Players were sold over the five years 1996–2001 for about NOK 90M, the golden age of Norwegian football. Nevertheless, the club was relegated in 2001. The books showed a deficit of 8M NOK in the same year. The club was promoted the following year, now with posted losses of 11M NOK. The year after, the deficit was 5M NOK. In 2006, the club lost NOK 10M, and a NOK 14M in 2008 of turnovers at 68M (loss-to-turnover ratio of approximately 21%). The figures illustrate that an SBC syndrome was now gaining a solid foothold in the club.

##### Discussion of the hybrid organization model intervention

It was against a gloomy financial backdrop that the club and its close allies, represented by the local savings bank, local energy company, and a handful of local businessmen, decided to establish a joint-stock company structure to professionalize the commercial part of the club's operations (27). Included in the aforementioned strategic alliance was an already established distant benefactor. In addition, establishing the Dual Model was seen as an appropriate measure to attract external stakeholders to invest in player talents and infrastructure.

Table 5 shows that the underlying SBC syndrome did not become significantly harder with the introduction of the joint-stock company structure. Even after this intervention, significant normalized financial losses became more the rule than the exception in the years that followed the professionalization of the club's operations. According to the annual report for 2019, the limited company structure had accumulated NOK 104M in tax loss carried forward during the ten years of the limited company structure existence (see Table 5, Note 3). It, therefore, seems reasonable to conclude that the hybrid Dual Model structure could not harden the SBC syndrome already established under the previous pure membership club structure. In this study, therefore, the club structure does not seem to matter when it comes to the existence of an SBC syndrome at a professional top-tier football club.

#### Intervention 3: the national FFP regulations

Between 2000 and 2009, the revenues in top Norwegian football increased as more spectators attended the matches, and media and sponsorship revenues also rose sharply. The problem, however, was that costs increased even more (59). The SBC syndrome was repeated across Europe [e.g., (6)]. Eventually, the NFA realized that the clubs could not manage their finances. The association, therefore, introduced a standard financial follow-up system for the industry in 2009 (the so-called FOS system). This system raises red flags when the club cash balances move toward zero. In addition, financially severely irresponsible clubs are punished sportively through points deductions (60). In 2012, the UEFA introduced a similar system, FFP, for its Champions League and Europe League competitions (45). The UEFA requires that the national follow-up systems be harmonized with their own. Nevertheless, the discrepancies can be significant, and the regulations in UEFA FFP are, therefore, in practice, significantly stricter than in the softer Norwegian variant.

##### Discussion of the Norwegian FFP regulations

A prerequisite for rational financial decisions is that the information basis provides an accurate picture of operational realities. In addition, clubs should apply the same accounting principles over time to be comparable. In the nation in question, this was a problem (60). Among other things, third-party ownership was allowed until 2014, after first being banned in the English Premier League in 2008 and later by the European Parliament and FIFA in 2015 (61). TIL Spiller AS, established in 2009, was wound up accordingly in 2014. However, during the five years when this company existed, the consolidated accounts that formed the basis for the club license and the financial FFP assessment did not give a true and fair view of the club's finances. About 19M NOKs in off and write-downs of players were not reported to the Norwegian FFP body. The license and FFP flagging (see Table 6) were thus based on incomplete figures. It was consolidated figures from the club (sports team), and TIL Fotball AS that were provided to the association. Figures from TIL Spiller AS were not included because the NFA allowed such inadequate reporting practice. To be credible, costs related to the depreciation and write-downs of players should, of course, also have been part of the basis for the association's assessment of the club's finances. The flags in the FFP system were then lifted on the wrong premises. Thus, the soft financial management initiated by the NFA, to a certain degree, appeared as a ritual with limited practical value. Individuals and firms in the club's network still contribute to player purchases and to pay players’ salaries without being included in the consolidated club accounts that are reported to the Norwegian FFP. For example, in 2009, the media reported that the TILs goalkeeper was the highest-paid player at the club, with a salary of about EUR 400,000 a year. The club never hid that the salary was paid by an external partner that was a subsidiary of the local savings bank.

**Table 6:** FFP signaling, normalized operating costs, and league position.

	FFP signal*	Normalized Operating Costs	League Position
Before FFP Regulations			
2006	N/A	58	10
2007	N/A	71	6
2008	N/A	82	3
2009	N/A	91	6
Average		76	6
After FFP Regulations			
2010	R-R	86	3
2011	Y-Y	89	2
2012	Y-Y	99	4
2013	Y-G	98	15
2014	Y-G	62	18
2015	Y-Y	64	13
2016	Y-G	56	13
2017	G-R	54	11
2018	G-Y	44	10
2019	Y-Y	56	15
Average	R's = 3	71	10
	Y's = 12		
	G's = 5		

* R, Red light; Y, Yellow light; G, Green light. Source: https://www.fotball.no/contentassets/87df25d67ce24877bf82060455dcf0a0/klubblisens-mediapresentasjon-2019.pdf.

Unconvincing flag raising is another soft spot in the Norwegian FFP system. Even if the FFP decisions were based on a hard and thus accurate picture of the total club economy, the assessments’ criteria are also soft (60). This softness affects the validity of the flag raising. It is poor operations that, over time, create financial problems in a business. In an average year, therefore, the revenues from football activities should cover all costs associated with the same activities. However, operation accounts for just over a quarter of the total score in the Norwegian FFP system. Operations are thus strongly underweighted (60). Instead, the importance of donations from benefactors and deposits from owners is overestimated. Fast and short-term contributions from benefactors are welcomed, while the quality of operations is toned down in the national FFP system. The flagging also shows that a club can be close to bankruptcy in one 6-month period and be declared financially very healthy during the next 6-month period, and then end up loss making again the following period. Table 6 shows the years 2017/18 TIL was first in the green zone. Then the flag went red, and six months later, the flag was green again. Table 5 shows that normalized operating profit was negative for both these years but that the club received donations that greenwashed the finances according to the FFP reporting in the 2.5 years when these were received and posted.

##### Impact of the FFP intervention on the budget constraint syndrome

Table 6 shows that the club economy was in the red 2010. In the following years, the club had been in the green five times, in the yellow 12 times, and only once in the red. Based on the signaling of the national FFP system, it seems reasonable to conclude that the BC approach had hardened in the last decade. However, the normalized operating profit in Table 5 shows that the club did not achieve a break even position in a single year since the national FFP was introduced. However, normalized operating costs (the column in the middle of Table 6) dropped significantly after 2013. So did the club's league position.

Andreff (21) argues that the break-even rule in UEFA's FFP regime is too soft as it allows clubs to operate with significant deficits as long as they have secured funding for the losses. This study has thus discussed the existence of significant soft spots in the Norwegian equivalent of FFP. The gaps must be closed for the system to achieve credibility and legitimacy among the actors.

## Overall discussion

This historical in-depth case study explores the evolution of an SBC syndrome in the world's northernmost professional top-tier football club (that is, TIL) over several decades. The individual impacts on the SBC syndrome of significant budget interventions made by the club and its allies were discussed together with current findings independently in the previous chapter. This chapter will discuss the overall impact of the interventions on the club's SBC syndrome, provide theoretical and practical implications, and outline some limitations.

The shift in BC behavior of TIL from hard to soft was triggered initially by the gradual increased financial thresholds necessary to compete in the sport after the club was promoted to the top level of the football pyramid more than thirty years ago (27, 41). The promotion highlighted that a continued hard BC behavior could not maintain a sustainable sportive venture at that historical point. The new soft BC institution was thus founded on one that was successful in economic terms but was doomed to fail in sports (21).

The HBC institution initially contributed to economic and sportive sustainability. But in the face of a radically more cost-intensive competition arena, an institution based on competing on more or less equal financial terms failed (47). Money mattered more and more to the club's sportive competitiveness. The centuries-old high-quality HBC institution at TIL degraded to a low-quality SBC institution, especially in the decades after the turn of the millennium.

The overall objective of soft financial management of football clubs is to secure improved sportive success still combined with economic survival (16, 17, 21, 29, 32). In the case club TIL, significant interventions were introduced that directed the club's financial management towards an SBC syndrome. The chrono­logy of the interventions showed that the club's dominant BC syndrome changed from being relatively hard in the period before 2005 to becoming significantly softer after that. This change in BC syndrome was related to ongoing financial injections from a distant benefactor (Intervention 1: Benefactor support, also see Table 4). The transformation of the formal structure of the club from a membership organization to a combined membership and limited liability structure embracing soft equity was another significant intervention (Intervention 2: The Dual Model, also see Table 5). Implementing a national FFP system with significant soft spots could not turn the SBC tide.

A (BC) institution has the potential to affect a club's performance (46, 62). In 2007–2012, the club performed very well in sports (see Table 6, rightmost column) as it became more competitive due to soft funding (47). The club did, however, perform poorly financially as its economic stability was threatened (see Table 6, leftmost column).

The overall aim of the present study was to contribute to a better understanding of the long-term underlying drivers behind an SBC syndrome in a team sports club [e.g., (16, 17, 21)]. Thus, this historical analysis focuses on why an SBC emerged in an individual football club, how it expressed itself in terms of financial and administrative interventions, and finally, why it eventually became persistent and thereby institutionalized. The following section will discuss the potential contributions of the study in more detail.

### Potential contributions

#### Contribution 1: hard talk followed by a soft walk

First, the club's BC intentions (talk) were scrutinized in this study by analyzing the report from the club's Control Committee and the Investor prospectus (see Tables 1, 2). This information was presented when the commercial part of the top football club was transformed into a joint-stock company (the Dual Model) some fifteen years ago. However, the HBC intention expressed was not realized by the BC interventions made by the club and its allies. It was actually soft money that talked through continuous cash injections from a benefactor and soft equity deposits from the local bank and energy company.

To understand why and how an SBC syndrome evolves, it is essential to follow the cash flows of the actors and not the wording in festive speeches or official documents, according to the present study. The talk and the walk advanced in quite different BC directions, and the gap between the two grew ever more significant as the club drifted away from its original path. The rhetoric of the club hierarchy underlined the value of staying on an HBC path. Nevertheless, the BC walk materialized through significant BC interventions that added softness to the clubs’ BC approach. Eventually, the financial management of the club was unable to “shake free” of its soft economic history (64), and the SBC syndrome was taken for granted and became institutionalized (63). This finding implies that we must primarily study clubs’ behavior and not their rhetoric to gain better insight into which BC approach they follow. To the best of this study's knowledge, the evolution of the gap between hard BC talk and soft BC walk and its consequences has been only modestly addressed in past SBC research.

#### Contribution 2: financial regulations could not hinder a soft walk

Economically sustainable business operations are contingent on core activities achieving a break even position. Accordingly, the flag-raising of an FFP framework should be strictly adapted to HBC principles (21). Raising credible warning flags requires that all football-related costs and only football-related revenues are included in the basis for the flag raising. The Norwegian FFP is exposed to several soft spots. One such soft spot was the lack of a break-even rule (60). Moreover, the flagging should focus more on profitability than liquidity, not the other way around. As long as the club had liquidity to pay its bills, it was good to go according to the FFP system, irrespective of who provided the cash (ibid.).

Furthermore, player transfer losses and eventual profits were posted in the accounts of a third-party company (TIL Spiller AS). This firm did not have to report its financial records to the national FFP system. Consequently, a fake picture of the club's finances was presented and assessed by the national FFP authorities (ibid.). In this way, the club could partly bypass the regulations (45). This finding supports the critics of soft financial regulations (21) and is regarded as a contribution of the present study.

An imminent proposal is not to accept that clubs post donations as legitimate revenues or cost-reducing items in the financial reports submitted to FFP bodies. Such a rule change could give a solid signal to the field of football that soft funding of over­spending from benefactors is considered illegitimate. It is attractive for clubs to accept donations from benefactors because it can give them a sportive competitive advantage or prevent a corresponding disadvantage. However, regulators should not accept that soft donations provide a false impression of healthy club economies. In the long run, it is reasonable to believe that the competitive balance of the industry will benefit from clubs being managed based on an accurate financial picture of the core activities of the football business.

Even though financial regulations matter (46; also see Table 6), this study demonstrates that Norwegian regulations (Intervention 3) were too soft to curb the slide towards a gradually more pronounced SBC syndrome at the case club TIL.

#### Contribution 3: bringing to the foreground the softness of the environment

This analysis further demonstrates that the origin of the gradual long-term development towards a soft BC syndrome arose both from outside (Intervention 1: Benefactor support) and inside the football club (Intervention 2: transforming to a hybrid organization model) and also from a soft national FFP intervention (Intervention 3). Thus, the syndrome resulted from interactions between the club and its immediate environment. The two first interventions constituted different layers that directed the budget syndrome into an ever-softer path. The first, most basic, and long-lasting layer consisted of significant and continuous financial injections from a distant benefactor (Table 4). Most previous BC studies have focused only on the soft behavior of clubs (21). The environment in which the clubs operate has more or less faded in the background (6).

This study shows that the sub-optimal SBC syndrome was co-created by the club and its allies. If the alliance finds it legitimate to overspend to achieve the club's sporting ambitions, the management of the club will probably do so being more or less confident that the alliance partners at the end of the day are willing to softly finance the overspending (32). It seems unlikely that overspending will be curbed as long as the big spender (the club) and the soft funders all are part of the same strategic alliance which primarily pursues sporting goals.

#### Contribution 4: different degrees and mechanisms of softness

Storm et al. (26) request detailed studies of different mechanisms of softness and to identify varying degrees of softness in a different context. To the best of our knowledge, this is the first study within the literature of team sports economics that takes a long-term historical micro perspective on the development of an SBC syndrome. This case study shows why and how an SBC syndrome developed in the world's northernmost professional football club over more than thirty years. The findings reveal that layers of low-quality soft interventions were added to an initial HBC syndrome. Eventually, the club was locked into an ever-tightening SBC grip. Loosening the grip was challenging as it would put the club at a competitive sporting disadvantage (32, 46).

#### Contribution 5: has no European region escaped the SBC syndrome?

The case club TIL is exceptional in its unique geographical context with a cold and harsh arctic climate. The Arctic also has enormous distances and a very dispersed population. The Norwegian city Tromsø is located north of the Polar Circle and has about 75,000 inhabitants. The flight time to the capital, Oslo, in the south is about 1.5 h. From Tromsø, it is just as long a distance to the North Pole. At the outset, this physical environment may not be meant for professional football.

In contrast to many other countries that have discovered valuable natural resources such as oil and gas, Norway has shown great discipline in managing the funds from the extraction to benefit both current and future generations (64). An HBC culture may therefore be a characteristic of this country. However, such strict budgetary discipline seems absent in the case club studied here. The club is located at the outermost edge of Europe. As such, no geographical area of Europe seems to have escaped the SBC syndrome. This finding responds to Storm et al. (26), who call for more SBC studies in new empirical contexts.

#### Contribution 6: management implications

The practical objective of this article is to contribute to the sustainable financial management of team sports organizations such as football clubs. The focus has been on why an SBC syndrome occurred, how it happened, what characterized its development over time, and why it became persistent. A better understanding of the evolution of an SBC syndrome can hopefully aid a football club in keeping its BC walk better aligned with its BC talk.

Counterfactual, if the case club TIL had not accepted unconditional donations from its benefactor or refrained from converting the membership club into a hybrid model, it would have been affected by an SBC syndrome to a far lesser extent. But such strategic choices would also have inflicted on the club a significant sportive competitive disadvantage (6). Accordingly, the dilemma management faces when choosing between an HBC or SBC approach resembles a Gordian knot. To unlock the knot, the football industry needs support from effective rules that support an HBC syndrome so that the financial competition for the clubs becomes more even (32, 46).

### Limitations

The present paper is a case study with all the limitations inherent in this research format regarding generalizing the results. Furthermore, the study has focused on three extensive and strategically noteworthy interventions that all influenced the BC syndrome of the case club TIL. However, the interventions operated as an intertwined package, and it is difficult to isolate the impact of each intervention. Of course, many other factors in and around a football club can affect its propensity to follow an SBC approach. For example, the media can motivate the club to overspend, and so can the attitudes and behavior of senior management. Finally, the board must be willing to put its foot down when poorly reasoned and costly proposals arrive at the table.

This study further argues that the relationship between the economic performance of the club in the last decade and the introduction of low-quality financial interventions in the decade before was hardly accidental. It is reasonable to assume that these interventions also were causally related. In this historical analysis, the HBC syndrome (up to 2000) is compared with the subsequent period of a soft SBC syndrome. In the first, i.e., the HBC period, none of the financial interventions discussed in this paper were introduced. All three interventions were at work in the second period (the SBC period). Accordingly, the HBC period is an implicit counterfactual to the SBC period. Nevertheless, in real-world social studies, one must be cautious with claims of causality and, as already emphasized, the generalization of findings from a single case study.

## Data Availability

The original contributions presented in the study are included in the article/Supplementary Materials, further inquiries can be directed to the corresponding author.
